# Complete genome sequences of two novel autographiviruses infecting a bacterium from the *Pseudomonas fluorescens* group

**DOI:** 10.1007/s00705-017-3419-9

**Published:** 2017-05-27

**Authors:** Grzegorz Nowicki, Karolina Walkowiak-Nowicka, Agata Zemleduch-Barylska, Anna Mleczko, Patryk Frąckowiak, Natalia Nowaczyk, Emilia Kozdrowska, Jakub Barylski

**Affiliations:** 1 Department of Molecular Virology, Institute of Experimental Biology, Faculty of Biology, Adam Mickiewicz University, Umultowska 89, 61-614 Poznań, Poland; 2 Department of Animal Physiology and Development, Institute of Experimental Biology, Faculty of Biology, Adam Mickiewicz University, Umultowska 89, 61-614 Poznań, Poland

## Abstract

In this paper, we describe two independent isolates of a new member of the subfamily *Autographivirinae*, Pseudomonas phage KNP. The type strain (KNP) has a linear, 40,491-bp-long genome with GC content of 57.3%, and 50 coding DNA sequences (CDSs). The genome of the second strain (WRT) contains one CDS less, encodes a significantly different tail fiber protein and is shorter (40,214 bp; GC content, 57.4%). Phylogenetic analysis indicates that both KNP and WRT belong to the genus *T7virus*. Together with genetically similar Pseudomonas phages (gh-1, phiPSA2, phiPsa17, PPPL-1, shl2, phi15, PPpW-4, UNO-SLW4, phiIBB-PF7A, Pf-10, and Phi-S1), they form a divergent yet coherent group that stands apart from the T7-like viruses (sensu lato). Analysis of the diversity of this group and its relatedness to other members of the subfamily *Autographivirinae* led us to the conclusion that this group might be considered as a candidate for a new genus.

**Electronic supplementary material:**

The online version of this article (doi:10.1007/s00705-017-3419-9) contains supplementary material, which is available to authorized users.

The *Pseudomonas fluorescens* group includes bacteria commonly found in soil, fresh water, and seawater. Its members can be used to control plant diseases and are well known for their growth-promoting properties [1]. On the other hand, these microorganisms are also involved in food spoilage [2, 3].

To date, there are at least 293 sequenced phages infecting members of the genus *Pseudomonas,* eight of which infect bacteria from the *P. fluorescens* group [4].

In this paper, we describe two novel phages infecting the *Pseudomonas* strain GL3, which was isolated during earlier studies from Lake Góreckie, located in Wielkopolska National Park (Western Poland) [5, 6]. Based on the sequence of marker genes (16S rRNA, gyrB, and rpoB), we unambiguously assigned this bacterial strain to belong to the above-mentioned group but were unable to classify it at the species level.

Phages infecting strain GL3 were isolated independently from the same region (Wielkopolska Province, Poland): the first one from sediments of a park pond in Śrem, and the other from silt of the Warta River, collected in Poznań (near the influx of treated sewage from the city’s left-bank treatment plant) in the summer of 2014. Phage isolation was a part of a student scientific project; therefore, the name of the first phage (KNP) is an acronym for the Student Scientific Society in Polish. The second name (WRT) is an abbreviation of the sampling site where the phage was found. Phage particles were purified from infected lysates according to “Protocol: CsCl phage prep” by the Center for Phage Technology, Texas A&M University (available at https://cpt.tamu.edu/wordpress/wp-content/uploads/2011/12/CsCl-phage-prep-08-17-2011.pdf). Phage genomic DNA was extracted from the purified phage particles using a QIAamp DNA Mini Kit according to the manufacturer’s instructions.

Genomes of both phage isolates were sequenced using an Illumina MiSeq at Genomed SA (Warsaw, Poland). After removal of the adapter sequences, reads were quality trimmed and randomly subsampled with Trimmomatic GPL v3 [7] and BBDuk v35.82 (http://jgi.doe.gov/data-and-tools/bbtools/) to obtain libraries with sizes corresponding to ~300 times the expected genome size. Prepared libraries were assembled using Geneious 9.1.6 (the software reported coverages of 289.8× for KNP and 296.8× for WRT) [8], MIRA 4.0 (261.0× KNP, 264.6× WRT) [9], Velvet Optimiser 1.2.10 (181.4× KNP ×128.8 WRT) [10], Edena v3_131028 (284.3× KNP, 290.8× WRT) [11] and SPAdes v3.9.0 (39,1× KNP, 41.6× WRT) [12]. The combination of the five different tools allowed us to cross-validate different assemblies. Additional verification was performed by mapping the raw reads back to each genome (we obtained mean coverages of 1209.3× for KNP and 2547.1× for WRT using the Geneious read mapper with medium settings). The obtained mapping was also used to determine the physical termini of both genomes (based on the read arrangement analysis with the Pause pipeline, available at https://cpt.tamu.edu/computer-resources/pause).

Protein-coding genes were predicted using GeneMarkS v4.32 [13], Glimmer 3.02 (iterative training) [14], PRODIGAL v2.6.3 [15], MetaGeneAnnotator v2008/8/19 [16], and ZCURVE_V (ZCURVE package 3.0) [17]. tRNA genes were predicted using tRNAscan-SE [18] (though none were found). Again, predictions generated with the different programs were compared, and CDSs identified by only a single tool with no BLAST hits against the RefSeq database were disregarded. Conflicting start codons were resolved based on majority voting of the prediction algorithms (which included BLAST hits). BLASTx alignments, together with conserved domains detected by InterProScan [19], were used to assign functions to protein products of the predicted genes. Both gene arrangements and functional annotations were subjected to detailed manual curations that included BLASTp searches against multiple databases (nr, RefSeq, UniProtKB), domain localisation (InterProScan, CD-Search [20]), ribosome binding site inspection, and literature review. Finally, PHIRE ver.1.00 [21] was employed to detect conserved phage regulatory elements.

The KNP and WRT genomes are composed of single linear DNA molecules with direct terminal repeats of 219 bp. Their lengths are 40,491 bp (KNP) and 40,214 bp (WRT), with GC contents of 57.3% and 57.4%, respectively. The two sequences are very similar (they share 97.1% identity in MAFFT [22] comparison; algorithm FFT-NS-2, scoring matrix 200PAM/k = 2, gap open penalty 1.53 and offset value 0.123), and their organisation is typical for genomes of autographiviruses. The left arm of each genome encodes predominantly proteins involved in DNA replication, while the right arm harbours genes involved in particle assembly. We predicted 50 CDSs in the KNP genome, all on the same strand. Putative functions were assigned to 28 of the CDSs (56%), while 22 (44%) had no known function. The gene arrangement in the WRT genome is virtually the same, although CDS 14, encoding a hypothetical protein with unknown function, is missing (Fig. 1). Almost all predicted genes have homologues in other viruses, mainly autographiviruses infecting *Pseudomonas* spp. CDS 3, present in both genomes, is the only gene that has no similarity to any known viral or bacterial sequences. Most other differences between these genomes can be described as single-nucleotide polymorphisms. However, the region encoding the C-terminus of the tail fibre and the following gene with unknown function (Fig. 1) vary significantly between analysed phages (the similarity drops below 60% over the 1-kb stretch of DNA).

**Fig. 1:**
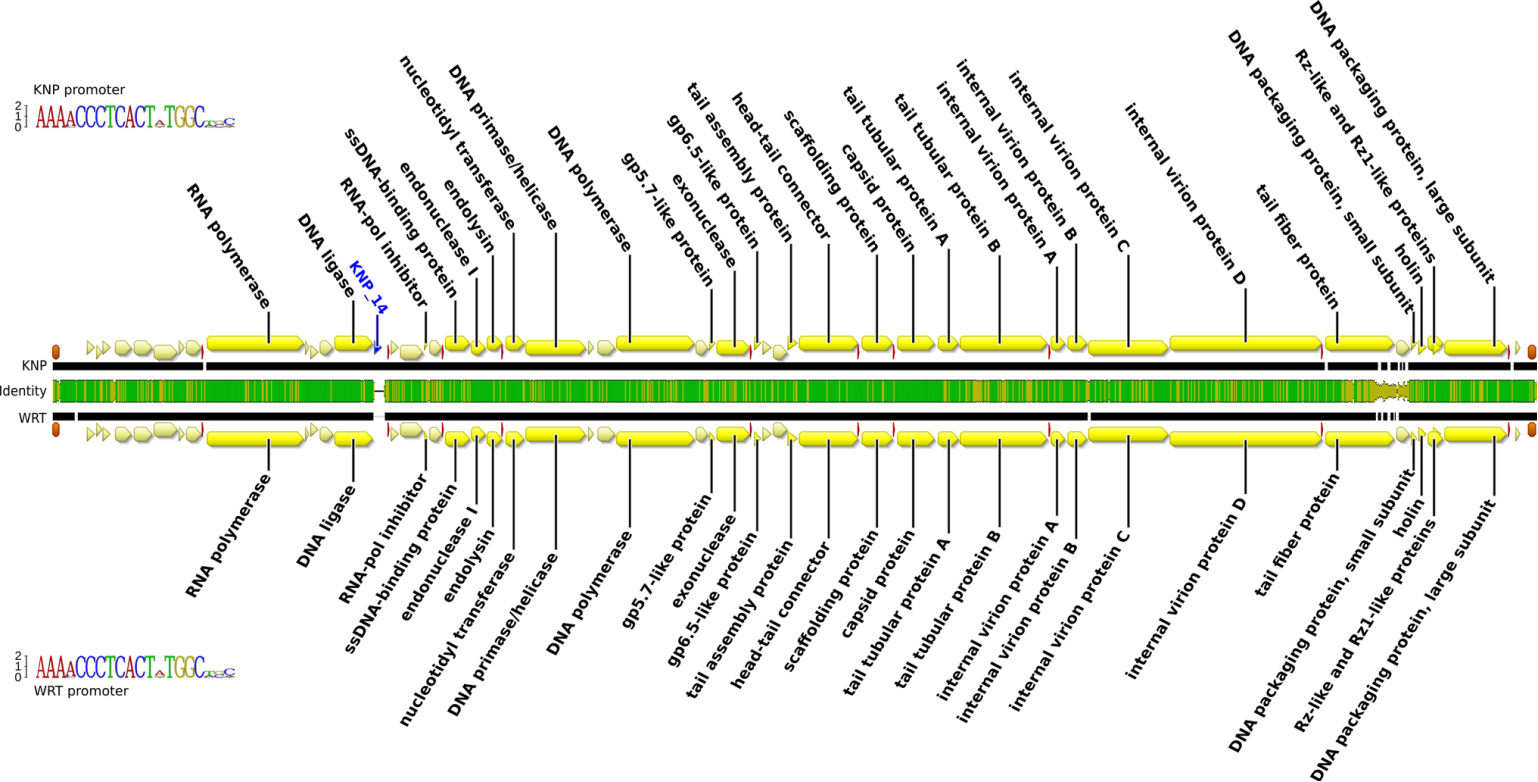
Genome map of bacteriophages KNP and WRT, shown as a pairwise alignment. Arrows indicate predicted genes (yellow) and promoters (red). Brown bars represent repeat regions. The middle bar shows DNA sequence similarity between the two genomes and is coloured from green (100% identity) through yellow (~50% identity) to red (less than 10%). Regions with no alignment are shown as a thin black line. Sequence logos shown next to each genome represent the consensus sequence of the phage promoter (color figure online)

Both phages are genetically similar to *Pseudomonas* phages phiPsa17 (KNP 93.1% and WRT 93.9% identity) and gh-1 (both 87.0% identity), which belong to the genus *T7virus* [4]. International Committee on Taxonomy of Viruses (ICTV) guidelines recommend DNA sequence identity of 95% as a threshold for species delineation. Thus, we report phages KNP and WRT as two isolates of the same candidate species, which we would like to name “*Pseudomonas virus KNP*”. Our phylogenetic analysis (see Fig. 2 and Supplementary file S1) supports the inclusion of this species in the genus *T7virus*. Careful examination of the results (discussed in detail in supplementary file S1 [23–27]) indicate that this genus is much more diverse than other currently approved genera in the subfamily *Autographivirinae*. Thus, we would like to suggest that this taxon could be split and that the formation of a new genus, “*Gh1virus*” (clustering T7-like *Pseudomonas* phages), might be considered.

**Fig. 2:**
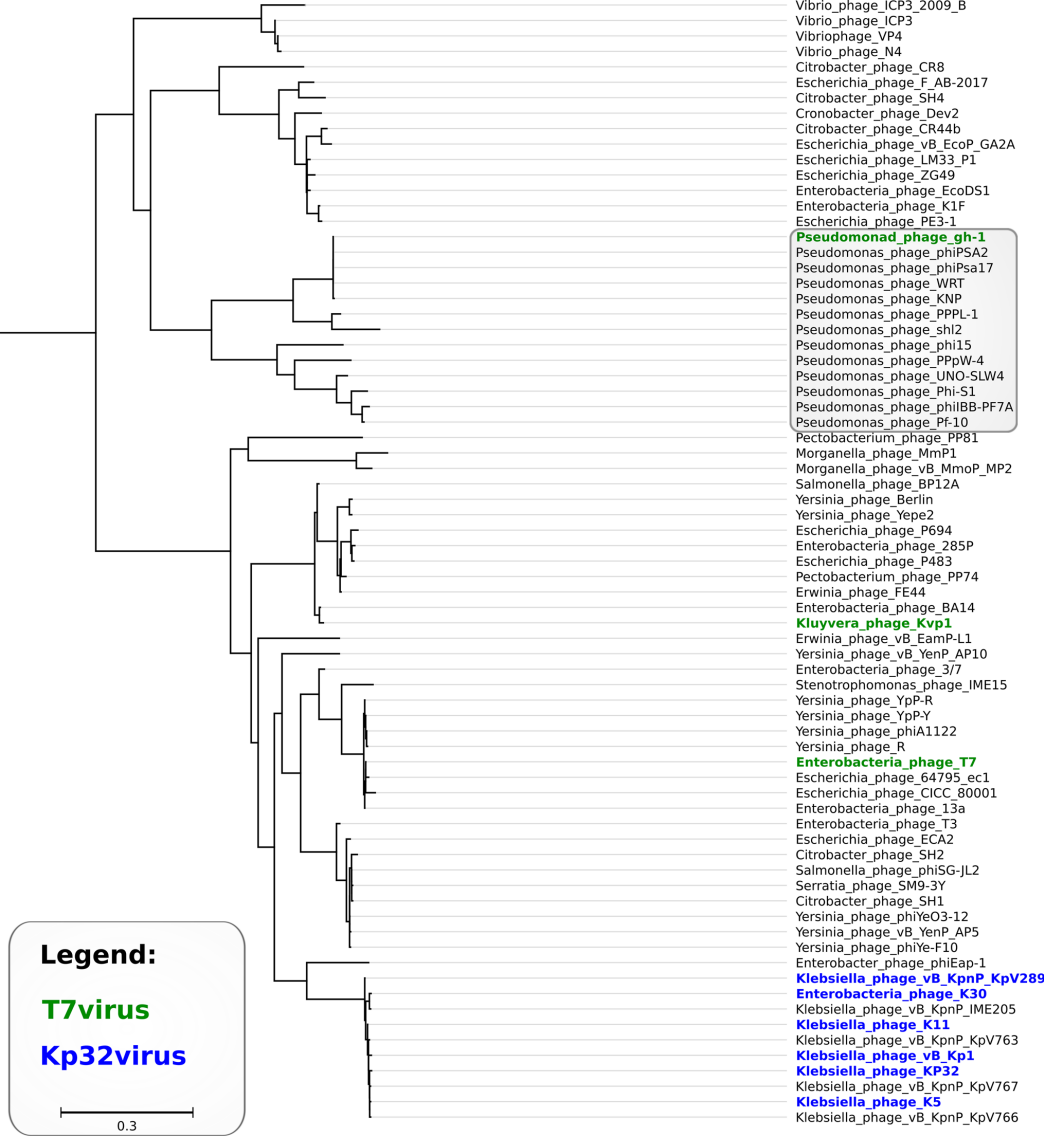
Approximately-maximum-likelihood tree based on the alignment of packaging ATPases – the sub-tree comprising T7viruses and Kp34viruses. Colouring (explained in the legend) represents ICTV-recognized genera. The light grey frame shows members of the gh-1 cluster

**Nucleotide sequence accession numbers**

The complete genomes of the phage KNP and WRT have been deposited in the NCBI database under the GenBank accession numbers KY798121 and KY798120, respectively.

## Electronic supplementary material

Below is the link to the electronic supplementary material.

Supplementary material 1 (PDF 1210 kb)

Supplementary material 2 (XLS 56 kb)
