# Supplementary material for: Complete genome sequences of two novel autographiviruses infecting a bacterium from the Pseudomonas fluorescens group
Source: Arch Virol. 2017 May 27;162(9):2907–11. doi: 10.1007/s00705-017-3419-9 (PMC5563517; doi:10.1007/s00705-017-3419-9)
Supplement: Supplementary file 1 — Supplementary material 1 (PDF 1210 kb) [file 705_2017_3419_MOESM1_ESM.pdf]

Complete genome sequences of two novel autographiviruses infecting a bacterium from the *Pseudomonas fluorescens* group

Supplementary file 1 – Phylogenetic analyses

To evaluate the phylogenetic positions of described phages and to propose their taxonomic classifications, we conducted an analysis of the genetic markers typically used to analyse Podoviridae phages: large subunits of DNA packaging ATPase (terminase) and viral RNA polymerases. Homologous protein sequences from other phages were retrieved by searching the nr database with BLASTp. We used the proteins mentioned above from KNP and WRT as queries and adopted an E-value cut-off 1e-10. After the search, hit sequences were carefully reviewed to remove redundant and erroneous records. We also discarded sequences with no associated taxonomic information (e.g., proteins from metagenomes and uncultured phages).

A search for packaging ATPases yielded a set of 194 unique sequences, all originating from podoviruses, specifically members of the subfamily *Autographivirinae*, or unclassified phages, *Acinetobacter* phage vB_AbaM_IME200 and *Klebsiella* phage vB_KpnP_KpV766, reported as myoviruses, are genetically nearly identical to established podoviruses and are misclassified. In the case of RNA polymerases, we found 217 unique sequences. Alike the packaging ATPases, these sequences were predominantly proteins from *Autographivirinae* phages. It is however worth mention that the results included some hits against phages from the *Siphoviridae* family, namely all members of genus *Xp10virus* and the lone type species of genus *Cbastvirus*.

After the curation of each set, we aligned its members using ClustalW (with default parameters) [23], refined the obtained alignments using MUSCLE [24] and selected suitable protein evolution models with ProtTest 3.4 [25]. The chosen model (in each case WAG+G) was used to generate approximately maximum-likelihood trees with FastTree 2.1.7 [26].

Generally, the groupings we obtained remained in agreement with the current release of the ICTV taxonomy, classification from NCBI databases, and literature data. Notable exceptions include the *Pantoea* phage LIMElight. This virus is classified to genus *Phikmvvirus*, but the topologies of trees based on both markers clearly indicate its closer relationship with *Kp34viruses*. This phylogeny is likely a result of the recent incomplete split of the *Phikmvvirus* [27] group. This rearrangement formed genus *Kp34virus* in its present form and left some orphan species still classified with the old taxon. Careful analysis of the obtained dendrograms suggest that the genus *T7virus* may also require a split in the near future, as the heterogeneity of this group significantly exceeds the diversity of the other currently accepted genera. The process of its division seemingly begun with the recent creation of the *Kp32virus*. It is notable that soon, a new genus clustering T7-like *Pseudomonas* phages might need to be created. As these viruses are prototyped by the *Pseudomonas* virus gh1, we suggest that the formation of the genus gh1virus might be considered.

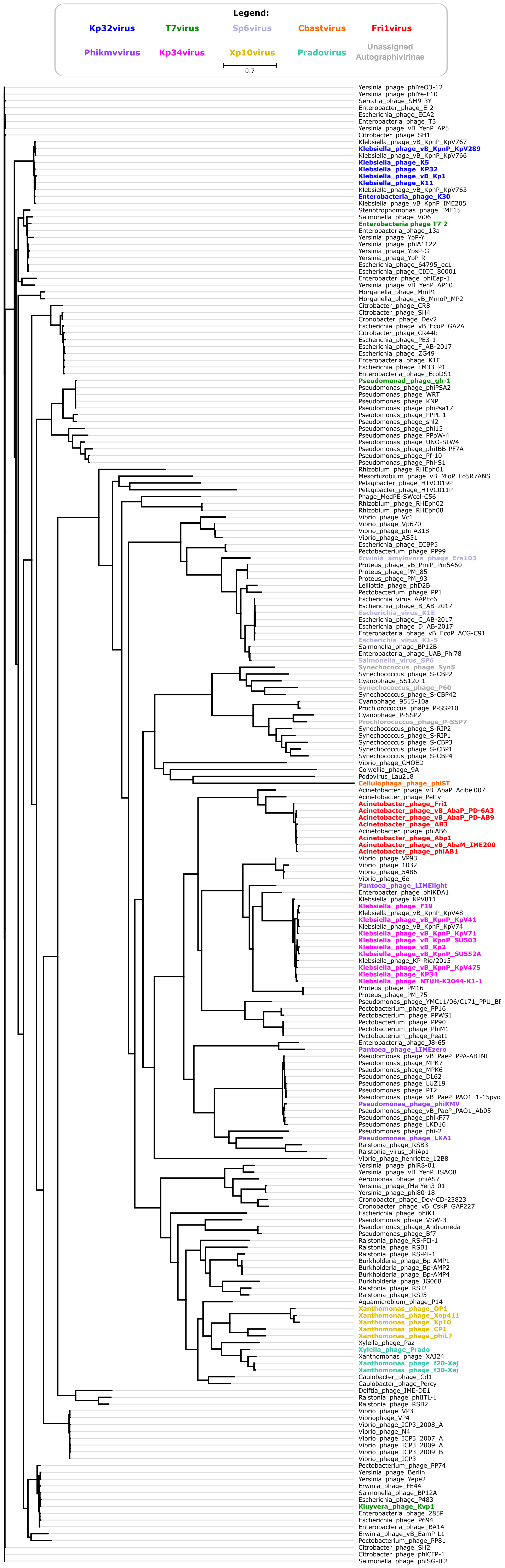

Fig. S1. Approximately-maximum-likelihood tree based on the alignment of of RNA polymerases. Colouring (explained in the legend) represents ICTV recognized genera

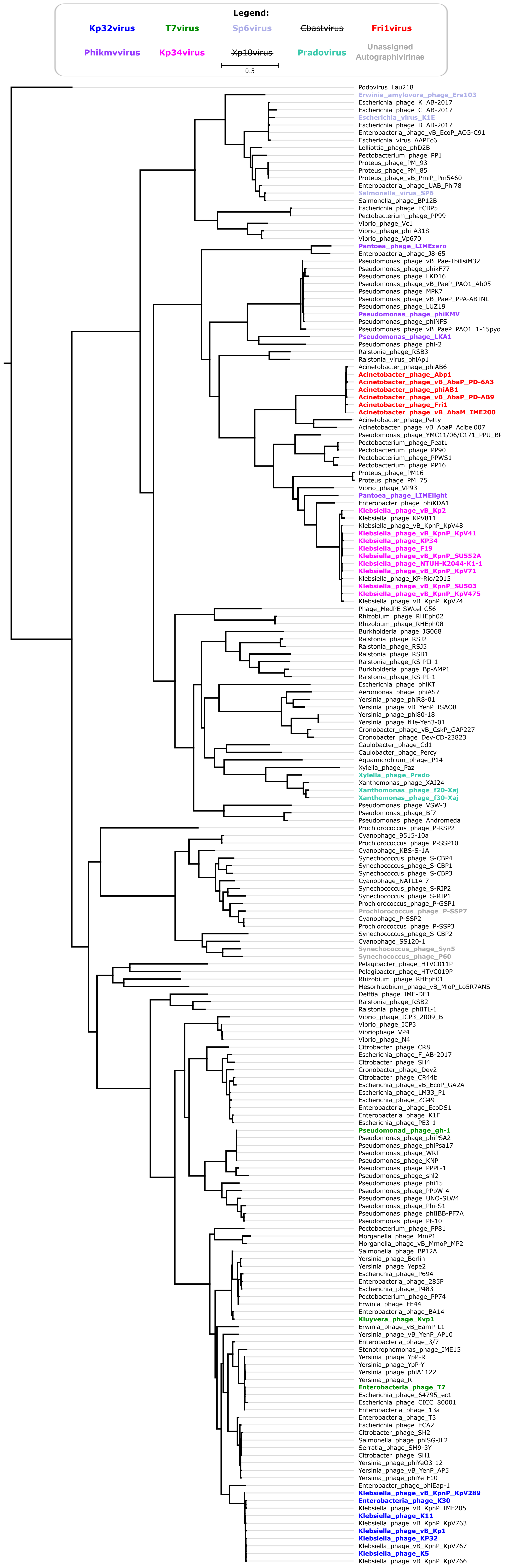

Fig. S2. Approximately-maximum-likelihood tree based on the alignment of DNA packaging ATPases. Colouring (explained in the legend) represents ICTV recognized genera
